# A mighty tool not only in perception: Figure-ground mechanisms control binding and retrieval alike

**DOI:** 10.3758/s13414-022-02511-5

**Published:** 2022-05-24

**Authors:** Philip Schmalbrock, Christian Frings

**Affiliations:** Department of Psychology, University of Trier, Universitätsring 15, DE-54296 Trier, Germany

**Keywords:** Figure-ground segmentation, Action control, Distractor-response binding, Motor control

## Abstract

Stimulus and response features are linked together into an event file when a response is made towards a stimulus. If some or all linked features repeat, the whole event file (including the previous response) is retrieved, thereby affecting current performance (as measured in so-called binding effects). Applying the figure-ground segmentation principle to such action control experiments, previous research showed that only stimulus features that have a figure-like character led to binding effects, while features in the background did not. Against the background of recent theorizing, integration and retrieval are discussed as separate processes that independently contribute to binding effects (BRAC framework). Thus, previous research did not specify whether figure-ground manipulations exert their modulating influence on integration and/or retrieval. We tested this in three experiments. Participants worked through a sequential distractor-response binding (DRB) task, allowing measurement of binding effects between responses and distractor (color) features. Importantly, we manipulated whether the distractor color was presented as a background feature or as a figure feature. In contrast to previous experiments, we applied this manipulation only to prime displays (Experiment 1), only to probe display (Experiment 2), or varied the figure-ground manipulation orthogonally for primes and probes (Experiment 3). Together the results of all three experiments suggest that figure-ground segmentation affects DRB effects on top of encoding specificity, and that especially the retrieval process is affected by this manipulation.

**Supplementary Information:**

The online version contains supplementary material available at 10.3758/s13414-022-02511-5.

## Introduction

The execution of the simplest action might seem all too trivial to us but, in fact, several processes contribute to even simple actions. Often summarized under the term *action control*, these sets of mechanisms allow humans to intentionally interact with their environment. One of these mechanisms is the binding of stimulus and response features (S-R binding).

The core assumption of S-R binding approaches in action control is that when responding to a stimulus, features of both response and stimulus are *integrated* into a short-lived episodic memory trace, called the *event file*, which comprises binary interconnections between features (Hommel, 2004). The *Theory of Event Coding* (TEC; Hommel et al., 2001) assumes that this is made possible through a common representational format (*common coding* assumption; Prinz, 1997), coding perceptual and motor information in the same code format. The assumed benefit of this integration process is the creation of a coherent representation of an action and its goal (action plan) that is protected against interference from other bindings (Stoet & Hommel, 1999).

Importantly, repeating some or all features in an event file leads to the *retrieval* of the whole event file from the previous episode, which improves or hampers performance. The retrieved event file might then influence current behavior and leads to performance costs or benefits. Costs and benefits are together referred to as S-R binding effects.^1^ The duality of integration and retrieval is captured by the *Binding and Retrieval in Action Control* framework (BRAC; Frings et al., 2020) that conceptualizes both processes as independent from each other and assumes that they can be further modulated by top-down (e.g., task instruction; Memelink & Hommel, 2013; Mocke et al., 2020) or bottom-up (e.g., salience; Schmalbrock et al., 2021) modulators individually.

Interestingly, it is assumed that S-R binding is not only a ubiquitous and automatic process, because it occurs almost inevitably (e.g., Hommel, 2005, 2009, 2019; Logan, 1988, 2002; but see Schöpper et al., 2020, for a limitation to this), but also that it incorporates a broad context because task-irrelevant stimuli are also integrated and can retrieve a previous event file (Frings et al., 2007). If S-R binding is indeed as broad as the literature claims, this would imply that not only irrelevant stimuli might be incorporated into an event file but also any irrelevant information like the background of an episode (Hommel, 2004). The present study investigates this relationship between action control and figure-ground segmentation.

### Figure-ground segmentation

Figure-ground segmentation is one of several *Gestalt principles*, a set of principles that are applied to incoming information (Wagemans et al., 2012, for a review). These kinds of principles guide how different aspects of incoming information are combined to form a coherent perception of objects that initially only exist as independent features. The basic idea is that through a defined set of principles that are applied to incoming information a relationship between basic features is imposed so that a coherent perception of an object can emerge (Vecera, 2000; Wischnewski et al., 2010). These principles can impose, for example, which elements of the scene “belong together” (grouping principles; Wertheimer, 1923), how to deal with occlusion of an otherwise coherent contour (contour integration; e.g., Elder et al., 2003), and what constitutes a background and what a figure in front of this background (figure-ground segmentation; e.g., Rubin, 1915).

What is perceived as background and what as the figure is determined by several external visual and internal cues that can be roughly grouped into two categories (see Wagemans et al., 2012, for a review): image-based principles like convexity or symmetry (Kanizsa & Gerbino, 1976), top-bottom symmetry (Hulleman & Humphreys, 2004), or lower region (Vecera et al., 2002), and non-image based influences like past experience (Peterson & Enns, 2005). These principles have been integrated into several modeling approaches that produce solid classifications of figure and ground (e.g., Domijan & Setić, 2008; Wischnewski et al., 2010). However, although we know much about how figure-ground segmentation emerges in the visual domain, we know less about how segmentation affects action control processes that use this visual information to produce an action that is contingent on the visual input.

### Action control and figure-ground segmentation

However, evidence from the figure-ground literature underlines that our cognitive system treads the background differently than a figure. For example, memory for background contours is substantially worse than for figure contours (Driver & Baylis, 1996) or discrimination accuracy for stimuli increases when presented on figures compared to backgrounds (Wong & Weisstein, 1982). Other research underlines that this processing disadvantage for background features probably emerges because the background receives considerably less attention than foreground figures (Mazza et al., 2005; Turatto et al., 2002). In these previous studies on the role of attention in figure-ground segmentation, a change detection task was used, where participants barely reported background changes when instructions were to report any change (compared to good figure change detection). Only when participants were instructed to solely focus on background changes did detection performance rise to foreground levels. They interpreted the fact that they had to specifically tell participants to focus on the background as evidence that attention had to be actively directed towards the background and that the “default mode” was to focus attention on figural features.

From an S-R binding perspective, these findings are rather intriguing. Previous studies showed that attention is a strong modulator of S-R binding (Moeller & Frings, 2014; Singh et al., 2018). Essentially, these previous studies found that attention to stimuli increased the S-R binding effect, while the absence of attention may even lead to the absence of binding. Take, for example, a study by Singh et al. (2018). They varied on which of two irrelevant (for the main task color discrimination) word dimensions participants focused on. Before a trial started, participants were informed which of two dimensions they needed to report after the trial. Intriguingly, S-R binding only emerged for the word feature that participants focused on due to the second task. Different from the literature on the automaticity of S-R binding, these two findings seem to suggest that S-R binding of background features should not be possible or should be at least greatly reduced.

This relationship between irrelevant background information and S-R binding effects has previously been investigated by Frings and Rothermund (2017). They presented participants with a distractor-response binding task (DRB; Frings et al., 2007). DRB is a sequential priming paradigm where participants are required to make an identity judgment towards a target stimulus in two consecutive displays – the prime and the probe display. Importantly, the target stimulus is presented alongside one or more distractor stimuli or features that repeat or change between prime and probe while the response to the target can also change or repeat.^2^ Repetition of the distractor can then retrieve the previous event file and with it the previous response, resulting in performance benefits when all features repeat but interference when only some but not all features repeat. Note that DRB effects are just another form of S-R binding effects where a distractor is the stimulus that is integrated with the response and upon repetition retrieves the previous response. Hence, we refer to the effects observed with the DRB paradigm as *DRB effects*.

In the study by Frings and Rothermund (2017), the irrelevant distractor was color. In one block, the distractor color was always presented as the background, while in the other block it was always presented as the figure in front of the background. Their results showed that color-response binding effects only occurred when the distractor color was a figure before the background, while DRB effects were absent when the distractor color was presented as the background.

Yet, the figure-ground manipulation was always applied to both the prime (where integration is assumed to play the major role) *and* the probe (where retrieval is assumed to play the major role for the occurrence of binding effects) displays. Since the BRAC framework (Frings et al., 2020) emphasizes that integration and retrieval are two independent processes, the previous manipulation makes it impossible to pinpoint whether one or both processes are actually affected by their figure-ground manipulation. Therefore, it is the goal of the present study to differentiate if and how this type of manipulation separately affects integration and retrieval.

### The present study

**Fig. 1:**
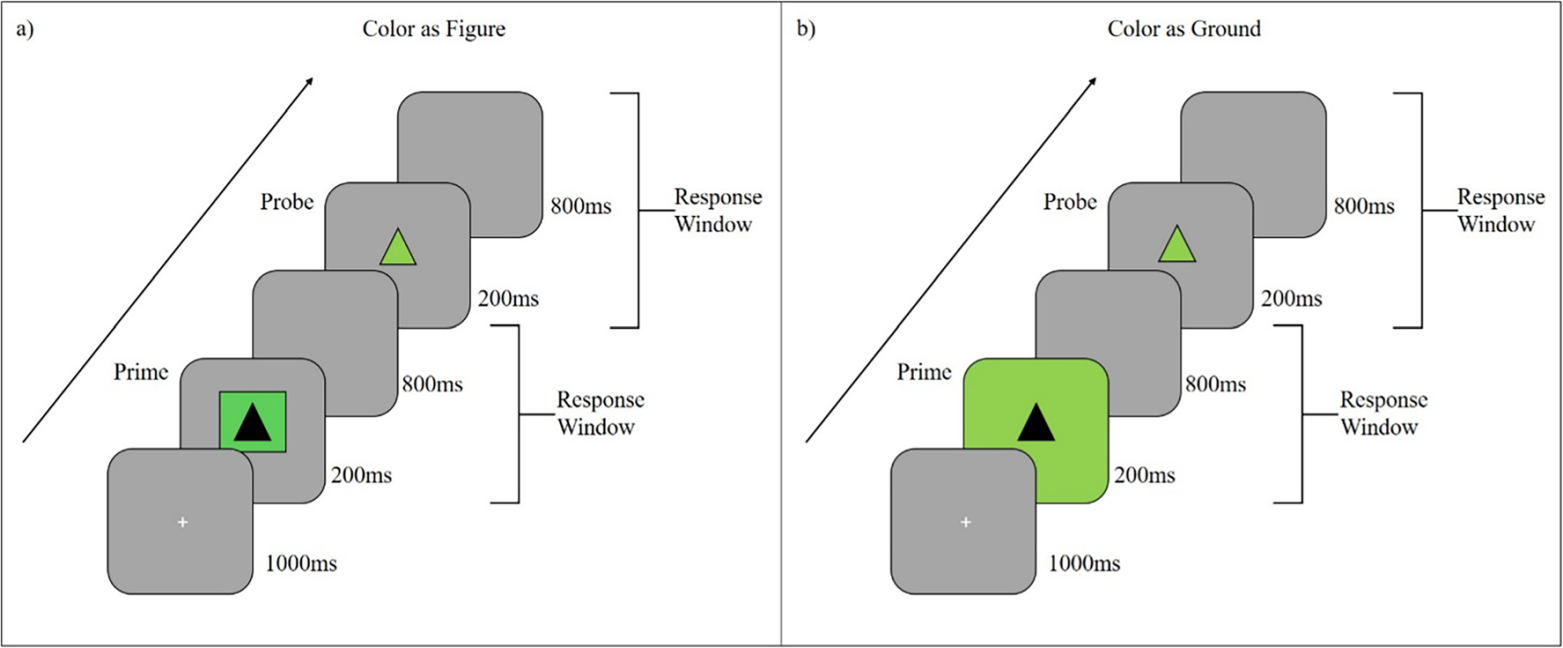
Exemplary experimental flow for the two prime layer configurations “figure” and “background.” (**a**) Figure layer configuration with response repetition and color repetition. (**b**) Background layer configuration with response repetition and color repetition. Responses were made towards the shape identity (triangle vs. square). Layer and probe target color (green vs. blue) was used as distractor. Color and stimuli are not drawn to scale

We adapted the DRB paradigm used by Frings and Rothermund (2017) so that the figure-ground manipulation was applied only to the prime (integration) or the probe (retrieval). We used the same figure-ground manipulation as the previous study by making the distractor an irrelevant color. To achieve segmentation, we presented this distractor color as either background or as a foreground figure. However, since we were interested in the influence of this segmentation manipulation on only integration and only retrieval, we applied it to only the prime (Experiment 1) or probe display (Experiment 2), respectively. We achieved this by presenting the distractor color as always part of the target stimulus in the display that was not subject to the present manipulation. For example, when applying the manipulation to only the integration, we present the distractor color as background or figure in the prime (the target would be an achromatic black shape) while the distractor color is always presented as part of the target in the probe (see Fig. 1). The same logic also applies to Experiment 2, except that we switched the figure-ground manipulation from prime to probe.

The figure-ground manipulation may play an important part in Experiment 1 (integration manipulation), because the cognitive system may divert attention only to foreground information. Since attention seems to be necessary for S-R binding effects (Moeller & Frings, 2014; Singh et al., 2018), this would mean that background features do not become integrated into the event file. Repeating or changing distractor colors in the probe would then have no consequences because there would be no distractor color in the event file that could be retrieved (and with them the whole event file).

The figure-ground manipulation may also play an important part in Experiment 2 (retrieval manipulation) because the retrieval process is strongly modulated by varying attention to features (Ihrke et al., 2011). If the processing of color distractors is diminished (or even extinguished) by our manipulation, this would have the consequence that even if distractor colors are part of the event file, they would not be retrieved due to the distractor features not being processed properly in the probe.

If integration or retrieval is affected by our manipulation, we should observe this through a significant interaction between response relation, color relation, and layer (background or figure) in Experiment 1 and/or Experiment 2 in reaction times (RTs) and/or error rates.

## Experiment 1: Figure-ground segmentation at the prime

### Method

#### Participants

Thirty students (data of one participant was lost due to technical error and replaced with a new participant) of Trier University (24 female; 29 right-handed) with a median age of 24.5 years (range 20–36 years) participated. All participants were tested for normal or corrected-to-normal vision. They gave written consent and received credits or payment of 10€ for their 1.15 h of service. This study was carried out according to the ethical standards defined by Trier University. The sample size was calculated according to previous studies investigating S-R binding effects, which typically led to medium-sized effects (*f* = 0.40). Thus, we planned to run *N* = 30 participants, leading to a power of 1 − *β* = 0.85 (assuming an alpha = 0.05; GPower 3.1.9.2; Faul et al., 2007), to observe the basic DRB effect without pinpointing the size of the assumed interaction. This would be the same sample size as the previous study that investigated this form of manipulation (Frings & Rothermund, 2017).

#### Design

For Experiment 1, three within-participant factors were varied: response relation (response repetition vs. response change), color relation (color repetition vs. color change), and layer (figure vs. background).

#### Apparatus and stimuli

Stimuli were presented on a 22-in. display monitor (60-Hz refresh rate, 1,680 × 1,050 pixels resolution) at a distance of approximately 50 cm. Before testing, the monitor was warmed up for at least 5 min to ensure temporal stability of luminance and color (Poth & Horstmann, 2017). A HP KU-0316 keyboard was used for response executions (QWERTZ layout). The experiment was programmed and run in Psychopy (Peirce et al., 2019; Version 03.01.2020).

Two distinct displays were presented (see Fig. 1): prime and probe display. In the prime display the target was either a triangle or a square (height: 0.8° × width: 0.8°) presented in dark grey (RGB_255_: 64, 64, 64; 46 cd/m^2^ ±1). The target was presented within a colored box (height: 1.95° × width: 1.95°, linewidth: 0.01°) or in front of a colored background. Background and box were colored either blue (RGB_255_: 128, 128, 192; 45 cd/m^2^ ±1) or green (RGB_255_: 51, 146, 51; 44 cd/m^2^ ±1), changing from trial to trial at random. In the probe display, a singular target was presented in either blue or green – repeating the prime color or changing to the unused color. When no colored prime background was presented (i.e., in the figure condition), a light grey tone (RGB_255_: 134, 134, 134; 41 cd/m^2^ ±1) background was presented instead.

#### Procedure

Participants were tested individually in a soundproof chamber. Instructions were presented on the screen. Participants were instructed to place their left index finger on the left arrow key and their right index finger on the right arrow key.^3^ It was emphasized that responses were to be made as fast as possible while maintaining high accuracy. A training with 20 trials was completed before the experimental block – participants received performance feedback after both prime and probe training trials. After the training finished participants only received feedback when they made an erroneous response. Additionally, participants were informed about their performance in the frequent breaks. They received feedback about the number of correct responses in the last 32 trials and their overall mean RT.

The task consisted of two consecutive responses. In both prime and probe participants had to classify the identity of the presented shape. If the shape was a triangle, they responded with the left arrow key; if the shape was a square, they responded with a right arrow key press.

The experiment consisted of 480 trials with a break after each 32-trial block. A single trial consisted of the following chain of events: A trial began with a fixation mark (+) presented at the screen center for 1,000 ms, followed by the prime. The prime display was presented for 200 ms with a response window of 1,000 ms, beginning with display onset. The prime always ended with the end of the response window (independent of the response) and was immediately followed by the probe display. The probe display was, again, presented for 200 ms with a 1,000-ms response window. Each trial was separated from the next by a blank screen for 1,500 ms.

The three factors response relation, color relation, and layer were varied orthogonally. In response repetition trials, the same response required in the prime was required in the probe. Vice versa, in response change trials a different response was required in prime and probe. In color repetition trials, the prime color was again presented in the probe. In color change trials, the prime color was different from the probe color. In figure layer trials, the prime target was presented in a small box colored in the distractor color. In background layer trials, no box was present and the whole background was presented in the distractor color.

### Results

Data processing and analysis were done with R (R Core Team, 2019; version 3.6.1). The package ‘dplyr’ (Wickham et al., 2019) was used for data processing and aggregation. Experimental conditions were compared using a repeated-measures analysis of variance (ANOVA) with type-III sums of square, using the ‘ezAnova’-function from the package ‘ez’ (Lawrence, 2016). We report three effect sizes for ANOVAs: *η*_*P*_^2^ and *η*_*G*_^2^ (Bakeman, 2005). The distractor-response binding effect is computed as the color repetition benefit in response repetition trials minus the color repetition interference in response change trials ([RRCC-RRCR]-[RCCC-RCCR]). This is another form of representing the two-way interaction between response relation and distractor relation. Note that the square root of the *F*-value (i.e., the *t*-value) for the critical three-way interaction as well as the *p*-value is equal to the *t*-value and *p*-value for the *t*-test comparing the DRB effects in both conditions. DRB effects were compared using post hoc *t*-tests complemented by Bayesian *t*-tests (Rouder et al., 2009), the Bayes factor (*BF*_01_) of which quantifies the evidence in favor of the null hypothesis relative to the evidence in favor of the alternative hypothesis. Bayes factors were computed using the package ‘BayesFactor’ (Morey & Rouder, 2018).

#### Data processing

Only RTs longer than 200 ms and shorter than 1.5 interquartile ranges over the third quartile of each person’s RT distribution were analyzed (see Tukey, 1977). Only probe RTs in trials with correct answers in both prime and probe were considered. According to these constraints, 11% of all trials were discarded. See Appendix Fig. 6 for a full plot of the RTs and error rates in each condition, and Appendix Figs. 7 and 8 for a plot of individual DRB effects.

#### Reaction times

**Fig. 2:**
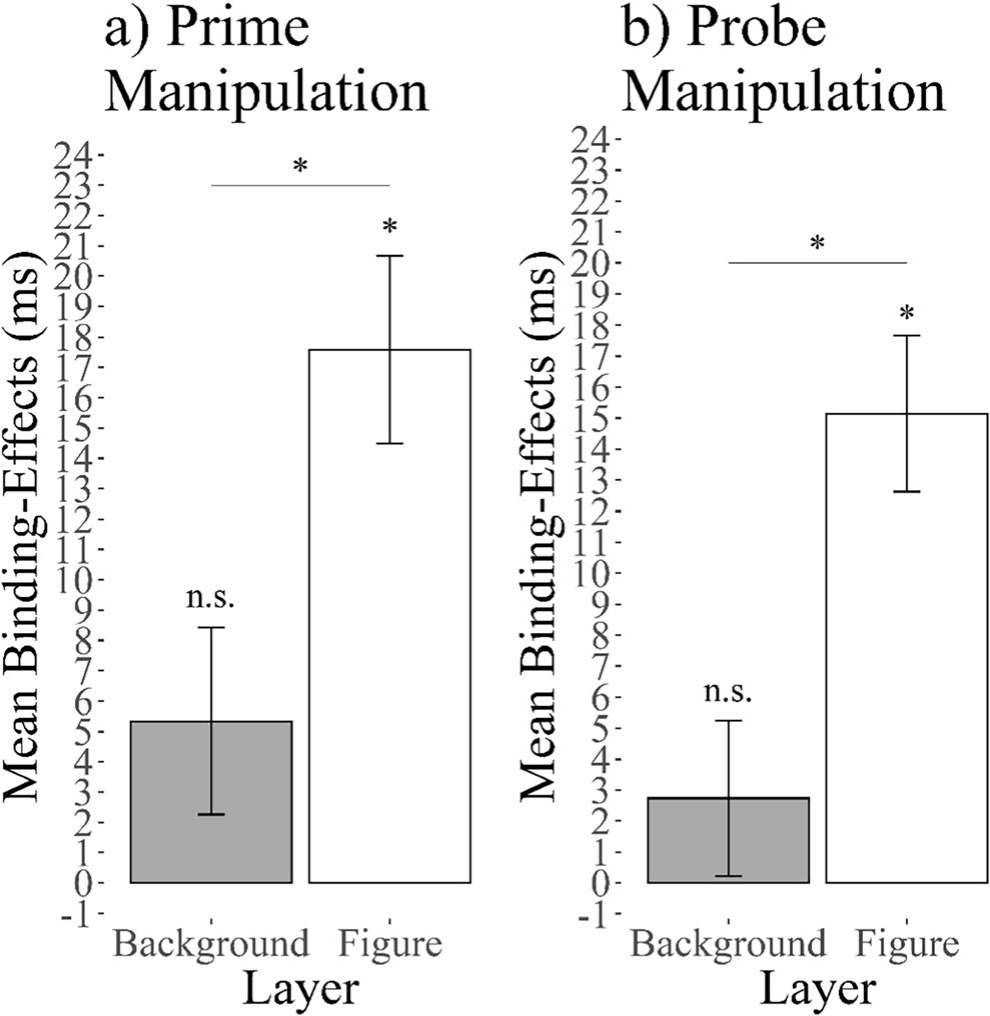
Average distractor-response binding (DRB) effects as a function of display layer for (**a**) Experiment 1, manipulation in prime, and (**b**) Experiment 2, manipulation in probe. *Note:* Error bars indicate within-participants error of the mean (Morey, 2008). See Appendix Figs. 7 and 8 for a plot of the individual binding effects

A 2 (response relation: repetition vs. change) × 2 (color relation: repetition vs. change) × 2 (layer: figure vs. background) repeated-measures ANOVA on probe RTs yielded a significant two-way interaction between response relation and color relation, *F*(1, 29) = 66.50, *p* < .001, η_*G*_^2^ < .01, *η*_*P*_^2^ = .70, indicating a significant DRB. Intriguingly, this interaction was further modulated by layer, resulting in a significant three-way interaction, *F*(1, 29) = 7.88, *p* = .008, η_*G*_^2^ <.01, *η*_*P*_^2^ = .21, suggesting that the DRB effect depends on the layer the prime distractor color was presented in. This effect is further supplemented when Bayes factors are considered: A paired *t*-test underlined that the DRB effect for the figure layer condition (*M* = 18 ms, *SD* = 13) was significantly different from the background layer condition (*M* = 5 ms, *SD* = 18), two-sided *t*(29) = 2.81, *p* = .008, *d*_*z*_ = 0.86, *BF*_01_ = 0.20 (see Fig. 2a). Post hoc analysis evidenced that the DRB effects were significantly different from zero for the figure layer condition (two-sided *t*(29) = 7.23, *p* < .001, *d*_*z*_ = 1.32, *BF*_01_ < 0.01) but not for the background layer conditions (two-sided *t*(29) = 1.94, *p* = .062, *d*_*z*_ = 0.36, *BF*_01_ = 0.99).

Additionally, several main and interaction effects were observed. A main effect for response relation was observed, *F*(1, 29) = 8.52, *p* = .007, η_*G*_^2^ = .01, *η*_*P*_^2^ = .23. Participants responded faster in response repetition trials (*M* = 384 ms, *SD* = 46) compared to response change trials (*M* = 398 ms, *SD* = 52). A main effect for layer was observed, *F*(1, 29) = 26.05, *p* < .001, η_*G*_^2^ < .01, *η*_*P*_^2^ = .47. Participants responded faster when the prime distractor color was presented in the background layer (*M* = 388 ms, *SD* = 50) compared to when the prime distractor color was presented in the figure layer (*M* = 394 ms, *SD* = 49). An interaction between response relation and layer emerged, *F*(1, 29) = 4.85, *p* = .036, η_*G*_^2^ < .01, *η*_*P*_^2^ = .14, where participants responded fastest in response repetition × background layer trials (*M* = 380 ms, *SD* = 46), somewhat slower in response repetition × figure layer trials (*M* = 389 ms, *SD* = 46), even slower in response change × background layer trials (*M* = 396 ms, *SD* = 52), and slowest in response change × figure layer trials (*M* = 400 ms, *SD* = 52). The main effect for color relation did not reach significance, *F*(1, 29) = 0.14, *p* = .709, η_*G*_^2^ < .01, *η*_*P*_^2^ < .01. The interaction between color relation and layer also did not reach significance, *F*(1, 29) = 2.65, *p* = .114, η_*G*_^2^ <.01, *η*_*P*_^2^ = .08.

#### Error rates

For the same analysis on probe error rates, only trials with correct prime responses but incorrect probe responses were considered (i.e., 4.40% of all trials were relevant error trials). The repeated-measures ANOVA on error rates yielded a significant interaction for response relation and color relation, *F*(1, 29) = 14.21, *p* < .001, η_*G*_^2^ = .03, *η*_*P*_^2^ = .32, again, indicating a DRB effect. This interaction was not further modulated by the layer the prime color was presented in, *F*(1, 29) = 2.42, *p* = .130, η_*G*_^2^ < .01, *η*_*P*_^2^ = .33. A paired *t*-test underlined that the DRB effect for the figure layer condition (*M* = 4 %, *SD* = 5) was not significantly different from the back layer condition (*M* = 2 %, *SD* = 5), two-sided *t*(29) = 1.56, *p* = .131, *d*_*z*_ = 0.40, *BF*_01_ = 1.75. Post hoc analysis evidenced that the DRB effect was significantly different from zero for the figure layer condition (two-sided *t*(29) = 3.73, *p* < .001, *d*_*z*_ = 0.68, *BF*_01_ = 0.26) but not for the background layer conditions (two-sided *t*(29) = 1.63, *p* = .115, *d*_*z*_ = 0.30, *BF*_01_ = 1.59).

Additionally, a main effect for layer emerged, *F*(1, 29) = 10.91, *p* = .002, η_*G*_^2^ = .04, *η*_*P*_^2^ = .27. No further main effect or interaction reached significance, all *F*s < 3.20 and *p*s > .130.

## Experiment 2: Figure-ground segmentation at the probe

### Method

#### Participants

Thirty new participants (one participant was excluded due to an excessive error rate above 25% of all trials, i.e., higher than the outlier criterion) of Trier University (24 female; 25 right-handed) with a median age of 23 years (range 18–32 years) were recruited for this experiment, following the same sample size calculation as the first experiment.

#### Design

For Experiment 2, three within-participant factors were varied: response relation (response repetition vs. response change), color relation (color repetition vs. color change), and layer (figure vs. background).

#### Apparatus and stimuli

Stimuli and apparatus were identical to the first experiment.

#### Procedure

**Fig. 3:**
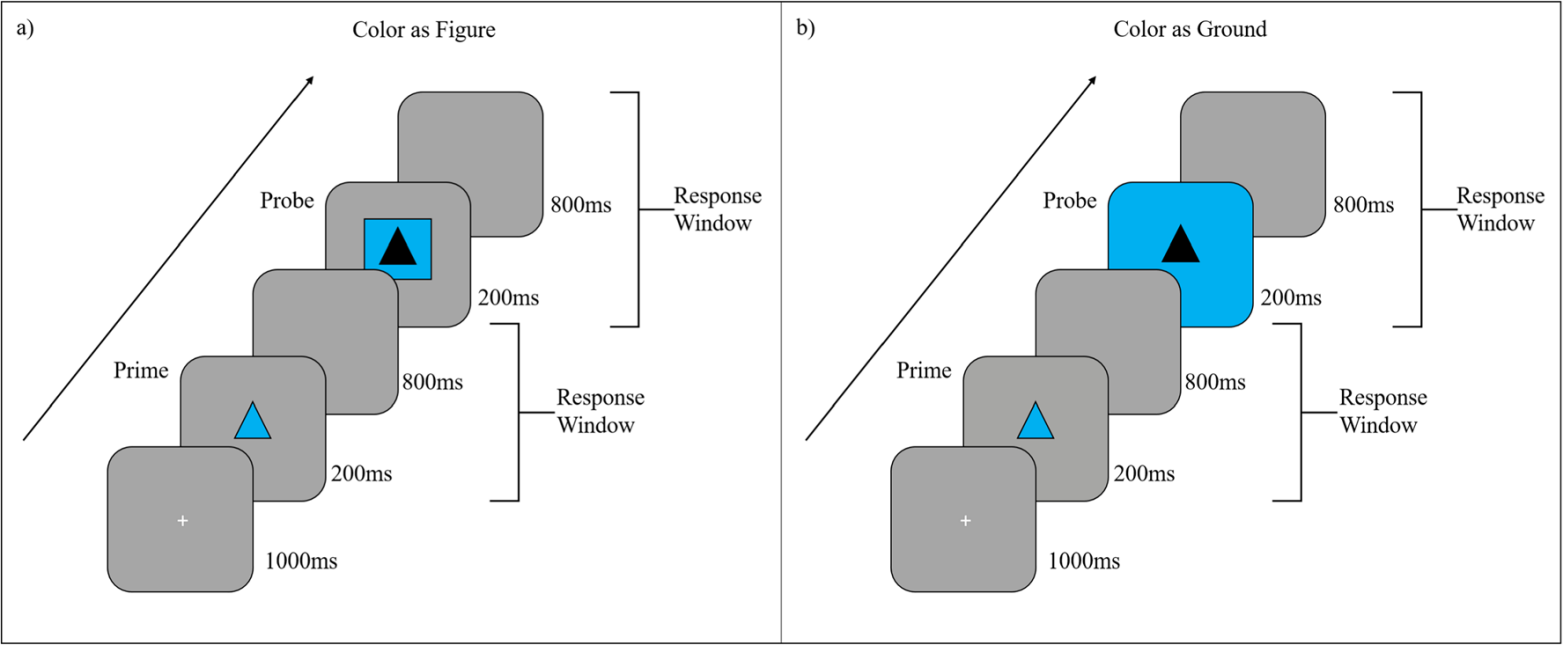
Exemplary experimental flow for the two probe layer configurations “figure” and “background.” (**a**) Figure layer configuration with response repetition and color repetition. (**b**) Background layer configuration with response change and color repetition. Responses were made towards the shape identity (triangle vs. square). Layer and prime target color (green vs. blue) were used as distractor. Color and stimuli are not drawn to scale

The procedure of Experiment 2 was nearly identical to the procedure of Experiment 1. However, now the probe distractor color could be presented as figure or background, and the prime stimulus was presented in the distractor color (see Fig. 3).

### Results

#### Data processing

Only RTs longer than 200 ms and shorter than 1.5 interquartile ranges over the third quartile of each person’s RT distribution were analyzed (see Tukey, 1977). Only probe RTs in trials with correct answers in both prime and probe were considered. According to these constraints, 12% of all trials were discarded. See Appendix Fig. 6 for a full plot of the RTs and error rates in each condition, and Appendix Figs. 7 and 8 for a plot of individual DRB effects.

#### Reaction times

A 2 (response relation: repetition vs. change) × 2 (color relation: repetition vs. change) × 2 (layer: figure vs. background) repeated-measures ANOVA on probe RTs yielded a significant two-way interaction between response relation and color relation, *F*(1, 28) = 14.27, *p* < .001, η_*G*_^2^ <.01, *η*_*P*_^2^ = .34, indicating a significant DRB. Intriguingly, this interaction was further modulated by layer, resulting in a significant three-way interaction, *F*(1, 28) = 13.35, *p* = .001, η_*G*_^2^ <.01, *η*_*P*_^2^ = .32, suggesting that the DRB effect depends on the layer the probe distractor color was presented in. This effect is further supplemented when Bayes factors are considered: A paired *t*-test underlined that the DRB effect for the figure layer condition (*M* = 15.24 ms, *SD* = 15.98) was significantly different from the back layer condition (*M* = 2.73 ms, *SD* = 15.26), two-sided *t*(28) = 3.65, *p* = .001, *d*_*z*_ = 0.84, *BF*_01_ = 0.03 (see Fig. 2b). Post hoc analysis evidenced that the DRB effect were significantly different from zero for the figure layer condition (two-sided *t*(28) = 5.04, *p* < .001, *d*_*z*_ = 0.94, *BF*_01_ < 0.01) but not for the background layer conditions (two-sided *t*(28) = 0.74, *p* = .468, *d*_*z*_ = 0.14, *BF*_01_ = 3.95).

Additionally, several main and interaction effects were observed. A main effect for layer emerged, *F*(1, 28) = 67.07, *p* < .001, η_*G*_^2^ = .02, *η*_*P*_^2^ = .71. Participants were faster when the distractor color was presented as the background (*M* = 396 ms, *SD* = 45) compared to the figure (*M* = 411 ms, *SD* = 42). Further, an interaction effect between response relation and layer emerged, *F*(1, 28) = 33.03, *p* < .001, η_*G*_^2^ <.01, *η*_*P*_^2^ = .54. Participants were fastest when the response repeated and the distractor was presented in the background (*M* = 391 ms, *SD* = 41), they were somewhat slower when the response changed and the distractor was presented in the background (*M* = 402 ms, *SD* = 49), they were even slower when the response changed and the distractor was presented as the figure (*M* = 409 ms, *SD* = 43), and were slowest when the response repeated and the distractor was presented as the figure (*M* = 413 ms, *SD* = 42).

#### Error rates

For the same analysis on probe error rates, only trials with correct prime responses but incorrect probe responses were considered (i.e., 5.24% of all trials were relevant error trials). The repeated-measures ANOVA on error rates yielded a significant interaction for response relation and color relation, *F*(1, 28) = 18.86, *p* < .001, η_*G*_^2^ = .04, *η*_*P*_^2^ = .38, again indicating a DRB effect. This interaction was not further modulated by the layer the probe color was presented in, *F*(1, 28) = 1.23, *p* = .277, η_*G*_^2^ < .01, *η*_*P*_^2^ = .04. A paired *t*-test underlined that the DRB effect for the figure layer condition (*M* = 4 %, *SD* = 7) was not significantly different from the back layer condition (*M* = 3 %, *SD* = 5), two-sided *t*(28) = 1.11, *p* = .277, *d*_*z*_ = 0.27, *BF*_01_ = 2.90. Post hoc analysis evidenced that the DRB effect was significantly different from zero for the figure layer condition (two-sided *t*(28) = 3.24, *p* = .003, *d*_*z*_ = 0.60, *BF*_01_ = 0.08) and for the background layer conditions (two-sided *t*(28) = 3.07, *p* = .005, *d*_*z*_ = 0.57, *BF*_01_ = 0.12).

Additionally, a main effect for layer emerged, *F*(1, 28) = 61.49, *p* < .001, η_*G*_^2^ = .20, *η*_*P*_^2^ = .69. Participants made less errors when the distractor color was presented as the background (*M* = 2.71%, *SD* = 3.08) compared to when the distractor was presented as the figure (*M* = 7.00%, *SD* = 5.91). An additional main effect for response relation also emerged, *F*(1, 28) = 8.06, *p* = .008, η_*G*_^2^ = .05, *η*_*P*_^2^ = .22. Participants made the least errors when the response changed (*M* = 3.08%, *SD* =3.70) compared to when it repeated (*M* = 5.90%, *SD* = 6.15). An interaction between response relation and layer was observed, *F*(1, 28) = 18.86, *p* < .001, η_*G*_^2^ = .07, *η*_*P*_^2^ = .40. Participants made the least errors when the response repeated and the distractor color was presented as background (*M* = 2.60%, *SD* = 3.20), made only marginally more errors when the response changed and the distractor color was presented as background (*M* = 2.82%, *SD* = 3.00), made substantially more errors when the response changed and the distractor color was presented as the figure (*M* = 4.80%, *SD* = 4.08), and made most errors when the response repeated and the distractor color was presented as figure (*M* = 9.20%, *SD* = 6.62).

No further main effect or interaction reached significance, all *F*s < 1.49 and *p*s > .231.

### Discussion

Experiments 1 and 2 suggest that background color features are neither integrated into an event file nor do they retrieve event files. This may underline not only the importance of distinguishing between integration and retrieval processes for the DRB effect but also may highlight that background features are indeed less prioritized for integration and retrieval processes than figure features.

However, for Experiments 1 and 2 it could be argued that there always is a mismatch between the prime layer and probe layer^4^ when DRB effects were diminished. That is, in the display that was not manipulated the distractor color was always presented as part of the target and was therefore always part of the figure. Thus, if the color feature was presented in the background in the manipulated display, the layers were mismatched. For example, if the color feature was presented in the background in Experiment 1 (prime only manipulation), it was always presented as the figure in the probe. An alternative interpretation of the data in Experiments 1 and 2 could therefore be not that background features are less integrated/retrieve less but rather that a layer mismatch leads to reduced DRB effects (possibly due to encoding specificity; Laub & Frings, 2020). To exclude this possibility, we orthogonally varied background layer in prime and probe.

For this new experiment, the encoding-specificity account would predict that instead of an effect of figure versus background, the DRB effects should order along the match versus mismatch that the figure-ground manipulation induces. That is, it would predict larger DRB effects in the two matching conditions (figure × figure and background × background) compared to the two mismatching conditions (figure × background and background × figure) but no difference between the two matching conditions – as only the mismatch would reduce DRB effects.

## Experiment 3: Figure-ground segmentation at prime and probe

### Participants

Sixty-four new participants of Trier University participated online. One participant was excluded due to not complying with the instructions (i.e., they did not respond in any trial). The final sample size consisted of 63 participants (51 female; 55 right-handed) with a median age of 21 years (range 18–35 years). In the absence of any previous study that investigated this kind of manipulation in this type of paradigm, we chose to double the sample size of the previous experiments (N = 60) plus four additional participants in case of drop-out due to the online setting, resulting in a planned sample of 64 participants.

### Design

For Experiment 3, four within-participant factors were varied: response relation (response repetition vs. response change), color relation (color repetition vs. color change), prime layer (figure vs. background), and probe layer (figure vs. background).

### Apparatus and stimuli

Stimuli and apparatus were identical to Experiments 1 and 2 with the following expectations. The experiment was run online and, therefore, was run on the local computer of each participant. Further, the letters D, F, J, and K were used as targets (each with a font size of 40 pixels).

### Procedure

Participants were recruited via the recruitment platform Sona (Sona Systems; sona-systems.com) and were then redirected to the online experiment platform Pavlovia (pavlovia.org) where they consented to participate via the online form. Instructions were presented on the screen. Participants were instructed to place their left index finger on the left arrow key and their right index finger on the right arrow key. If the target letter was an F or a D they responded with a left keypress, if the target was a J or a K they responded with a right keypress. It was emphasized that responses were to be made as fast as possible while maintaining high accuracy. A training period with 16 trials was completed before the experimental block – participants received performance feedback after both prime and probe training trials. After the training finished participants only received feedback when they made an erroneous response. Additionally, participants were informed about their performance in the frequent breaks. They received feedback about the number of correct responses in the last 32 trials.

The experimental block consisted of 480 trials with a break after each 32-trial block. A single trial consisted of the following chain of events: A trial began with a fixation mark (+) presented at the screen center for 1,000 ms, followed by the prime. The prime display was presented for 1,000 ms or until a response was registered. After the prime ended, a black screen was presented for 500 ms. Then the probe display was presented for up to 2,000 ms or until a response was registered. Each trial was separated from the next by a blank screen for 1,500 ms.

The four factors response relation, color relation, prime layer, and probe layer were varied orthogonally. In response repetition trials, the same response required in the prime was also required in the probe. Vice versa, in response change trials a different response was required in prime and probe. In color repetition trials, the prime color was again presented in the probe. In color change trials, the prime color was different from the probe color. In prime figure layer trials, the prime target was presented in a small box colored in the distractor color. In prime background layer trials, no box was present and the whole background was presented in the distractor color. In probe figure layer trials, the probe target was presented in a small box colored in the distractor color. In probe background layer trials, no box was present and the whole background was presented in the distractor color.

### Results

#### Data processing

Adhering to the same constraints as in Experiments 1 and 2, 22% of all trials were discarded.

#### Reaction times

**Fig. 4:**
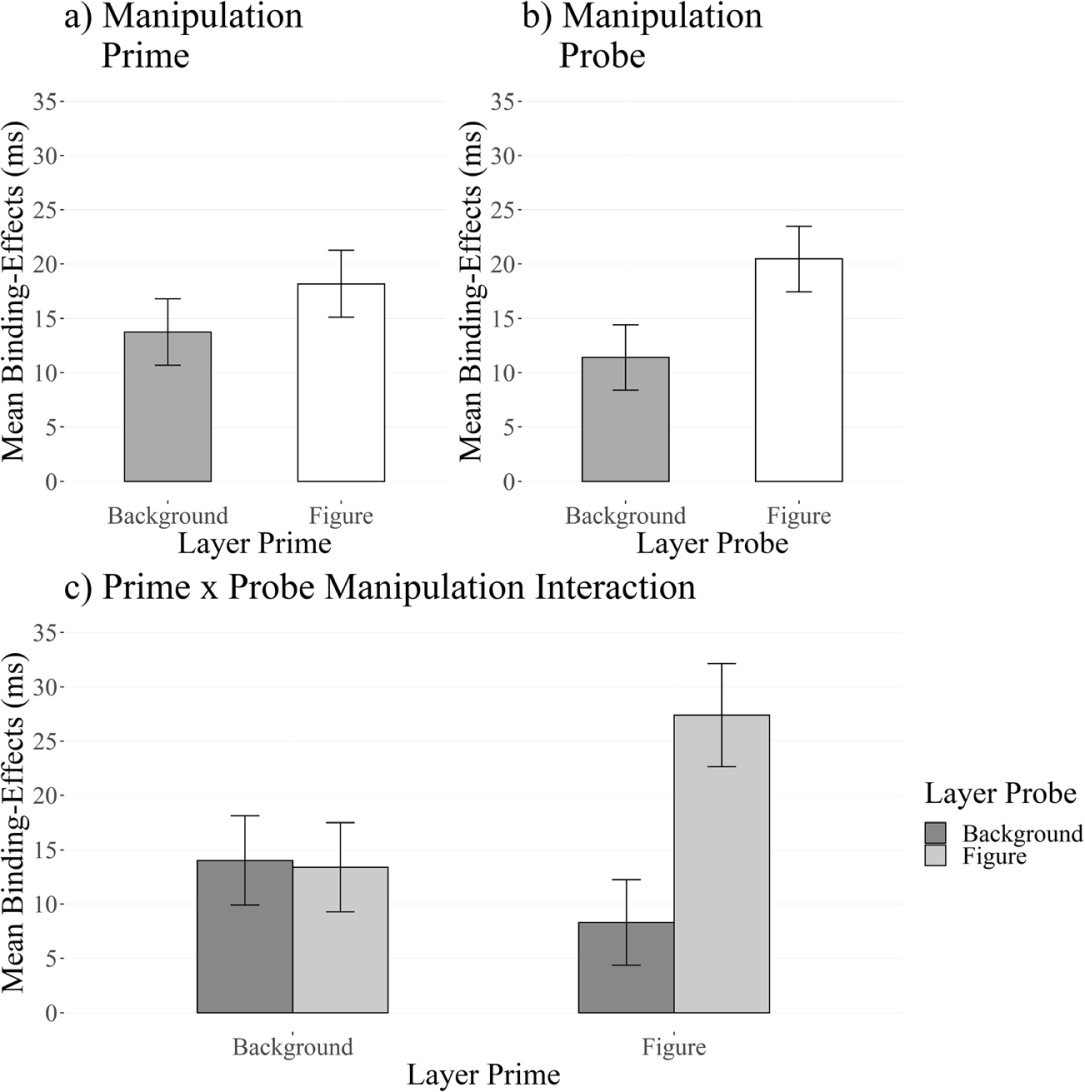
Average reaction time distractor-response binding (DRB) effects for Experiment 3. The two upper panels show the isolated effect of (**a**) only manipulating the prime and (**b**) only manipulating the probe. Panel (**c**) shows the interaction between prime and probe manipulation. Note that even when the color distractor is presented in the background in the prime and the probe, the DRB effects for this condition are still smaller than for the condition where the color distractor is presented as the figure in the prime and the probe. Error bars indicate within-participants error of the mean (Morey, 2008)

A 2 (response relation: repetition vs. change) × 2 (color relation: repetition vs. change) × 2 (prime layer: figure vs. background) × 2 (probe layer: figure vs. background) repeated-measures ANOVA on probe RTs yielded a significant two-way interaction between response relation and color relation, *F*(1, 62) = 40.80, *p* < .001, η_*G*_^2^ < .01, *η*_*P*_^2^ = .40, indicating the basic DRB effect. Further, a three-way interaction between response relation, color relation, and probe layer emerged, *F*(1, 62) = 4.61, *p* = .036, η_*G*_^2^ < .01, *η*_*P*_^2^ = .07. A post hoc *t*-test revealed that the DRB effect for the probe background layer (*M* = 11 ms, *SD* = 28) was significantly smaller than for the probe figure layer (*M* = 20 ms, *SD* = 23), *t*(62) = 2.15, *p* = .036, *d*_*z*_ = 0.27, BF_01_ = 0.88. DRB effects for probe background trials were significantly different from zero, *t*(62) = 3.25, *p* = .002, *d*_*z*_ = 0.41, BF_01_ = 0.07, as were DRB effects for probe figure trials, *t*(62) = 6.99, *p* < .001, *d*_*z*_ = 0.88, BF_01_ < 0.01. The three-way interaction between response relation, color relation, and prime layer was not significant, *F*(1, 62) = 0.95, *p* = .333, η_*G*_^2^ < .01, *η*_*P*_^2^ = .02. DRB effects for the prime background layer (*M* = 14 ms, *SD* = 27) were not significantly different from DRB effects for the prime figure layer (*M* = 18 ms, *SD* = 25), *t*(62) = 0.97, *p* = .333, *d*_*z*_ = 0.12, BF_01_ = 4.40. Interestingly, a four-way interaction between response relation, color relation, prime layer, and probe layer emerged, *F*(1,62) = 5.60, *p* = .021, η_*G*_^2^ < .01, *η*_*P*_^2^ = .08 (see Appendix Figs. 7 and 8 for a plot of individual DRB effects). Specifically, DRB effects in the prime background layer with probe background layer condition (*M* = 14 ms, *SD* = 39) were significantly smaller than DRB effects in the prime figure layer with probe figure layer condition (*M* = 27 ms, *SD* = 38), *t*(62) = 0.97, *p* = .333, *d*_*z*_ = 0.12, BF_01_ = 4.40. Intriguingly, the DRB effect in the prime background layer with probe background layer condition was not significantly different from the prime figure layer with probe background condition (*M* = 8 ms, *SD* = 32), *t*(62) = 1.04, *p* = .302, *d*_*z*_ = 0.07, BF_01_ = 4.33, nor from the prime background layer with probe figure layer condition (*M* = 13 ms, *SD* = 30), *t*(62) = 0.11, *p* = .913, *d*_*z*_ < 0.01, BF_01_ = 7.20 (see Fig. 4; please find all other comparisons in the Online Supplementary Material (OSM)).

#### Error rates

**Fig. 5:**
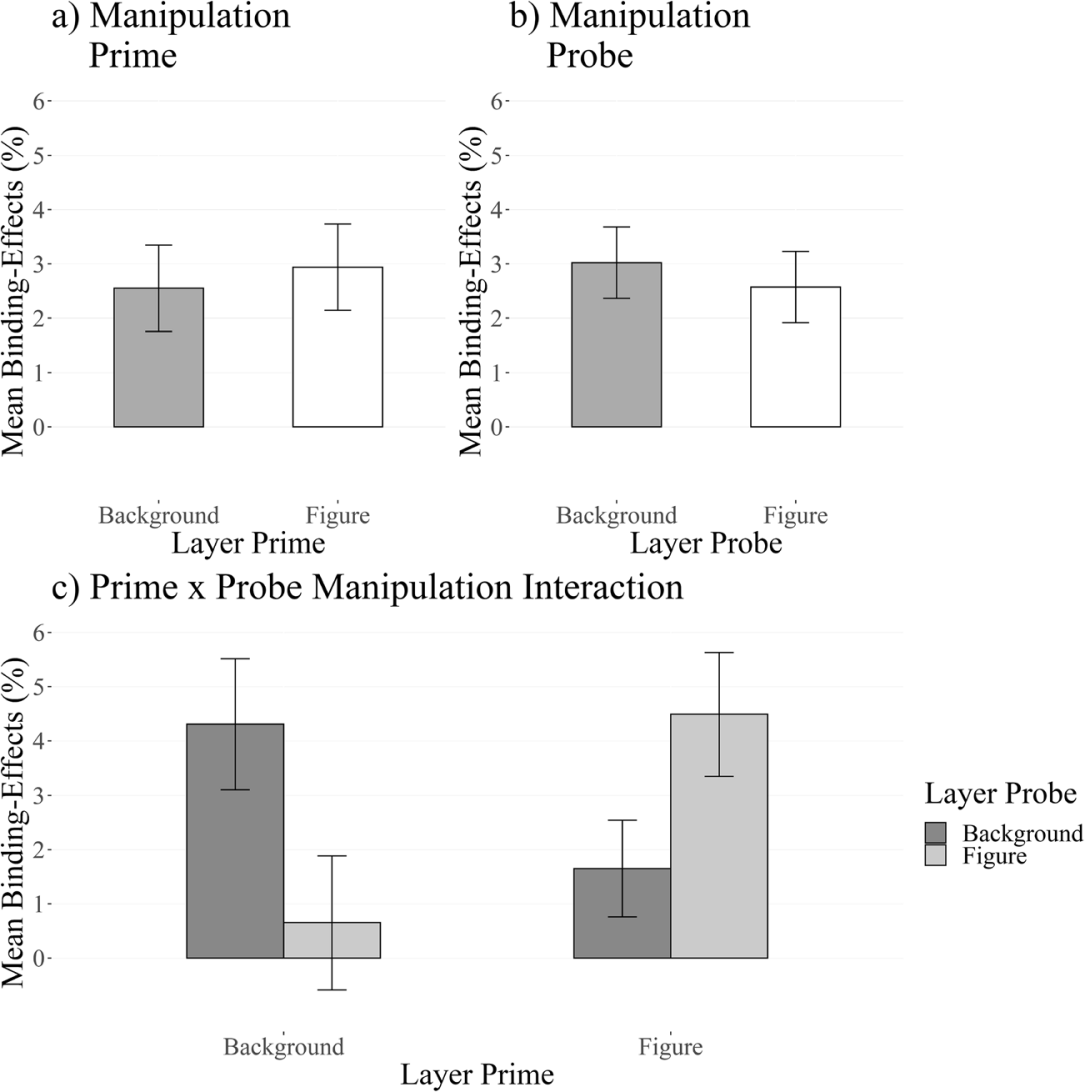
Average error rate distractor-response binding (DRB) effects for Experiment 3. The two upper panels show the isolated effect of (**a**) only manipulating the prime and (**b**) only manipulating the probe. Panel (**c**) shows the interaction between prime and probe manipulation. Error bars indicate within-participants error of the mean (Morey, 2008)

For the same analysis on error rates, 7% of all trials were relevant. This analysis yielded a significant two-way interaction between response relation and color relation, *F*(62) = 17.83, *p* < .001, η_*G*_^2^ = .01, *η*_*P*_^2^ = .22, indicating the basic DRB effect. Further, neither the three-way interaction between response relation, color relation, and probe layer, *F*(62) = 0.19, *p* = .659, η_*G*_^2^ < .01, *η*_*P*_^2^ < .01, nor the three-way interaction between response relation, color relation, and prime layer, *F*(62) = 0.25, *p* = .602, η_*G*_^2^ < .01, *η*_*P*_^2^ < .01, reached significance. Interestingly, the four-way interaction between response relation, color relation, prime layer, and probe layer reached significance, *F*(62) = 6.28, *p* = .015, η_*G*_^2^ < .01, *η*_*P*_^2^ = .09 (see Fig. 5). See the OSM for a full analysis of the DRB effects.

### Discussion

Experiment 3 further investigated an alternative explanation for the results presented in Experiments 1 and 2. That is, a mismatch between the prime and probe layer could also explain these findings based on the encoding specificity of the different displays. Therefore, in Experiment 3, the prime and probe layer were manipulated simultaneously, which could either produce a match between prime and probe layer or a mismatch between prime and probe layer.

First, if you just compare the conditions of Experiment 1 or Experiment 2 in Experiment 3, you see that we exactly replicated the previous results. Experiment 3 showed that, as in Experiments 1 and 2, a mismatch between prime and probe layer resulted in reduced DRB effects compared to the figure x figure condition.

As discussed above, these results coud be re-interpreted in terms of encoding specificity – a mismatch per se resulted in reduced DRB effects and not the figure-ground manipulation. However, a pure encoding specificity-based account would also assume that the matching background × background condition would also lead to strong DRB effects, which is not the case in Experiment 3. RT DRB effects in the background × background condition were significantly smaller than in the figure × figure condition and further did not differ from the DRB effects in the mismatching conditions. This suggests that the figure-ground manipulation has an effect on top of the encoding specificity. That is, DRB effects for background color distractors are reduced – even if prime and probe layer match. This indicates that although a mismatch between the prime and probe layer produces a reduced DRB effect, background colors are additionally less processed than figure colors. Encoding specificity can thus partially explain the results observed in Experiments 1 and 2 but figure-ground segmentation worked on top of the effect of encoding specificity.

We further looked at the isolated effect our manipulation had on prime and probe (i.e., the three-way interaction between response relation, color relation, and prime/probe layer). Here we only observed an effect of the figure-ground manipulation on the probe but not on the prime. Together with the four-way interaction, this indicates that especially retrieval is affected by the figure-ground manipulation.

## General discussion

In the present study, we investigated whether integration and/or retrieval in the DRB task is modulated by a figure-ground segmentation manipulation. A previous study showed that background color features did not lead to DRB effects *in general*. Yet, against the background of recent theorizing (Frings et al., 2020), we suggested that the two processes that contribute to DRB effects, integration and retrieval, might be affected independently by figure-ground segmentation manipulation.

In Experiments 1 and 2, we introduced a figure-ground manipulation to the DRB paradigm that only affected either prime (integration) or probe (retrieval). A distractor color was shown either as the whole background (background condition) or as its own figure in front of a background (figure condition). Overall, we found significant DRB as evidenced by the significant interaction between distractor and response relation. More importantly, we found that this DRB effect is further modulated by figure-ground segmentation as has been shown in previous studies (Frings & Rothermund, 2017). This was evidenced by the significant interaction between response relation, distractor relation, and display layer, as well as the Bayes factors favoring the alternative hypothesis. This effect pattern was observed in both experiments, however, note that we observed this pattern only in the RTs.

In Experiment 3, we investigated a possible alternative explanation in terms of encoding specifity for the results of Experiments 1 and 2. That is, a possible mismatch between the prime and probe layer may have caused reduced DRB effects in the background conditions of Experiments 1 and 2 because the distractor color was always presented as the figure in the not-manipulated display. However, although Experiment 3 revealed that encoding specificity affected the DRB effects, encoding specificity cannot fully account for the pattern we observed in the RT DRB effects.

Together Experiments 1–3 reveal a complex influence of figure-ground segmentation on DRB effects but also on integration and retrieval separately. First, the results reveal that encoding specificity can already emerge by just presenting a color dimension in different layers of scene. Thus, encoding specificity has to be considered for manipulations targeting prime and probe separately as even a slight mismatch (like features being presented in different layers of a scene) might affect the DRB effect. It is also noteworthy that figure-ground segmenatation can affect processing via encoding specifity. Second, in Experiments 1 and 2 we found reduced DRB effects for the background condition. That we found reduced DRB effects in the background × background condition suggests that the findings in the first two experiments cannot solely be attributed to encoding specificity. Thus, they can, albeit carefully, be interpreted as evidence that background features are indeed less likely integrated into an event file and are less likely to retrieve from an event file. However, in Experiment 3 we only found a significant effect of our figure-ground manipulation on the probe but not on the prime. This might suggest that especially retrieval is affected by the figure-ground segmentation manipulation. This would be well in line with previous findings in the literature that suggest that retrieval responds much stronger to manipulation than integration (Hommel et al., 2014; Ihrke et al., 2011).

In a sense, it could be argued that the figure-ground segmentation mechanism works as a gateway mechanism in action control, reducing the amount of irrelevant information that can interfere with the processes resulting in DRB effects. Arguing from a more outside-the-lab perspective, this intuitively makes sense. A unicolored background rarely contributes important information to our everyday actions. Since processing capacity is limited (Bundesen, 1990; Cowan, 2001), it makes sense to consider possible highly irrelevant information less for the competition of stimuli for the limited processing resources (Desimone & Duncan, 1995) and thus giving more important information a chance of processing. One caveat of many action control paradigms and surely the typical DRB paradigm is their very simple and easy to process displays; that is, oftentimes only one or two stimuli (e.g., two letters) are presented so that any kind of selection process may be irrelevant for these kinds of tasks. Thus, previous research might have underestimated the impact of selection processes like figure-ground segmentation. These processes may be much more important for action control in real-world scenarios that are much richer in information than in a laboratory setting.

Still, this consideration might not be generalized to all gestalt principles. At least for color grouping, it was also shown in the laboratory that integration and retrieval are modulated differently (Laub et al., 2018). Here, color-grouped prime distractor-target units led to stronger integration, but ungrouped probe distractor-target units led to stronger retrieval. Yet, it is important to emphasize that figure-ground segmentation and perceptual grouping contribute quite differently to our perception and have been observed to contribute to perception at different levels of processing (figure-ground segmentation as early as V1; e.g., Lamme, 1995, vs. grouping after binocular depth perception; e.g., Rock & Brosgole, 1964; although grouping may occur at different levels of perception; see Wagemans et al., 2012). While figure-ground segmentation may keep irrelevant information out of the cognitive system, grouping determines what belongs together – a process that does not require keeping information out of processing. Thus, it makes sense that both processes lead to different modulations of DRB effects because one hinders attentional deployment to specific features, while the other suggests what belongs together, a process that requires processing and attentional deployment to the features.

In conclusion, the present study extends previous findings by showing that our figure-ground segmentation possibly affected integration and retrieval processes separately. However, it was also revealed that encoding specificity plays an important role in this context. In the context of the figure-ground segmentation literature and the action control literature, the present study highlights the importance of attention for both processes and suggests that the cognitive bias for the foreground reduces the interference from irrelevant background information – not only in perception but also in action.

### Supplementary Information

ESM 1 (DOCX 78 kb)

## Figures and Tables

**Fig. 6:**
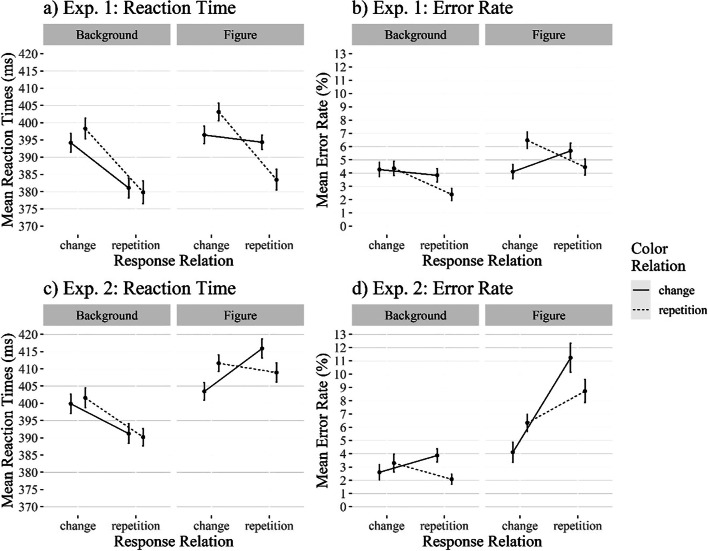
Mean reaction time and error rates as a function of response relation, color relation and layer for (**a**, **b**) Experiment 1 and (**c**, **d**) Experiment 2. Note: Error bars indicate within-participants error of the mean (Morey, 2008).

**Fig. 7:**
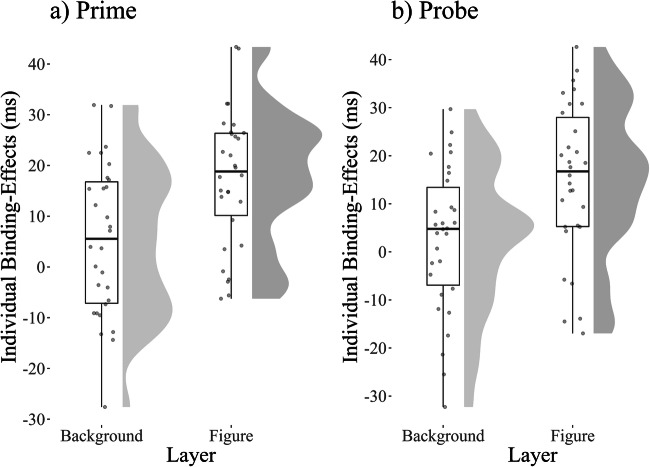
Raincloud plots (Allen et al., 2019) for individual binding effects as function of (**a**) prime layer (Exp. 1) and (**b**) probe layer (Exp. 2). The horizontal line in each boxplots represents the median of the distribution. Upper and lower whiskers extend to the largest / smallest value above / below the respective hinge but at most 1.5 times the interquartile range above and below the second and first quartiles (McGill et al., 1978)

**Fig. 8:**
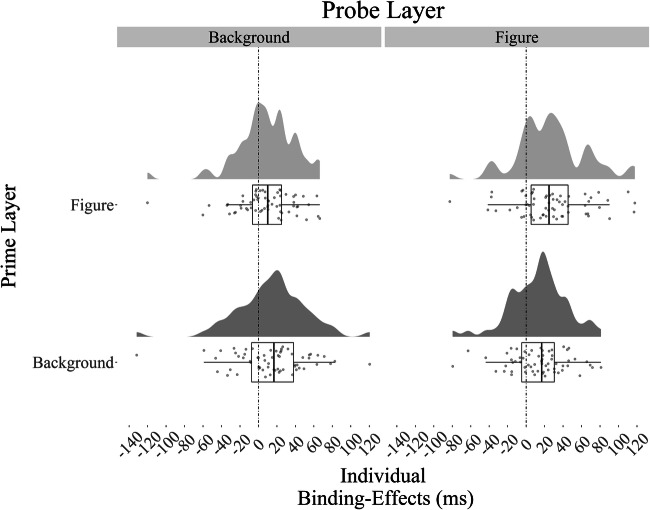
Raincloud plots (Allen et al., 2019) for individual binding effects as function of prime layer and probe layer for Experiment 3. The vertical line in each boxplots represents the median of the distribution. Upper and lower whiskers extend to the largest/smallest value above/below the respective hinge but at most 1.5 times the interquartile range above and below the second and first quartiles (McGill et al., 1978)

## Data Availability

The data that support the findings of this study (10.23668/psycharchives.5619), and the scripts for data processing and analysis (10.23668/psycharchives.4715) are openly available at “PsychArchives”. None of the experiments were preregistered.

## Appendix 1

### Mean performance in Experiment 1 (prime manipulation) and 2 (probe manipulation)

Plots show the average Probe RTs and Probe Error rates for Experiment 1 and Experiment 2 as a function of response relation (repetition vs. change), color relation (repetition vs. change), and layer.

## Appendix 2

### Individual reaction time binding effects for Experiment 1 and Experiment 2

Plots show the individual reaction time (RT) binding effects for each participant as a function of layer. Plots for (a) prime manipulation and (b) probe manipulation.

## Footnotes

1 Note that we use the term binding effect for the whole effect pattern that integration and retrieval contribute to (i.e., costs and benefits). This differs from some other studies that also used binding instead of integration or that used binding/integration interchangeably. We do this to avoid confusion between the formatting process of an event file (integration) and observed behavioral effects (S-R binding effect) that include the contribution of retrieval.

2 Distractor identity (color relation) and required response (response relation) can both independently repeat or change from prime to probe display. This leads to the four conditions: response repetition with color repetition (RRCR), response repetition with color change (RRCC), response change with color repetition (RCCR), and response change with color change (RCCC). Usually, full repetition trials (RRCR) lead to fastest reaction times (RTs) because the previous event file is retrieved, including the previous response, which can then be reused without having to compute a new response. Partial repetition (RCCR or RRCC) trials often lead to slower RTs because the repeating features retrieve the previous event file, which conflicts with the demands of the present episode – this conflict has to be resolved before response execution. Full chance trials (RCCC) elicit neither facilitation nor interference because no feature repeats that excludes the possibility for retrieval.

3 Please note that this 1:1 mapping does not allow us to disentangle the response relation from the target identity relation. However, previous findings (Frings et al., 2007, Exp. 2) showed that the target relation cannot fully account for the crucial interaction between response and distractor relation.

4 We thank an anonymous reviewer for pointing this out and suggesting the paradigm used in Experiment 3.

## Declarations

The research reported in this article was supported by the Deutsche Forschungsgemeinschaft (FR2133/14-1).
