# Supplementary material for: A mighty tool not only in perception: Figure-ground mechanisms control binding and retrieval alike
Source: Atten Percept Psychophys. 2022 May 24;84(7):2255–70. doi: 10.3758/s13414-022-02511-5 (PMC9481513; doi:10.3758/s13414-022-02511-5)
Supplement: Supplementary file 1 — (DOCX 78 kb) [file 13414_2022_2511_MOESM1_ESM.docx]

**Supplementary Material**

**Table 1**

*Results for the ANOVA on Reaction Times, Exp. 3*

| **Effect** | **Degrees of Freedom** | ***F*-Value** | ***p*-Value** | ${\boldsymbol{\eta}_{\boldsymbol{G}}}^{\boldsymbol{2}}$ | ${\boldsymbol{\eta}_{\boldsymbol{P}}}^{\boldsymbol{2}}$ |
| --- | --- | --- | --- | --- | --- |
| Response Relation | 1 \| 62 | 219.06 | < .001 | .10 | .78 |
| Color Relation | 1 \| 62 | 1.90 | .173 | < .01 | .03 |
| Prime Layer | 1 \| 62 | 14.73 | < .001 | < .01 | .19 |
| Probe Layer | 1 \| 62 | 1.44 | .235 | < .01 | .02 |
| Response Relation × Color Relation | 1 \| 62 | 40.80 | < .001 | < .01 | .40 |
| Response Relation × Prime Layer | 1 \| 62 | 0.05 | .829 | < .01 | < .01 |
| Color Relation × Probe Layer | 1 \| 62 | 0.03 | .858 | < .01 | < .01 |
| Response Relation × Probe Layer | 1 \| 62 | 0.02 | .888 | < .01 | < .01 |
| Color Relation × Probe Layer | 1 \| 62 | 0.32 | .574 | < .01 | .01 |
| Prime Layer × Probe Layer | 1 \| 62 | 9.74 | .003 | < .01 | .14 |
| Response Relation × Color Relation × Prime Layer | 1 \| 62 | 0.95 | .333 | < .01 | .02 |
| Response Relation × Color Relation × Probe Layer | 1 \| 62 | 4.61 | .036 | < .01 | .07 |
| Response Relation × Prime Layer × Probe Layer | 1 \| 62 | 33.75 | < .001 | < .01 | .35 |
| Color Relation × Prime Layer × Probe Layer | 1 \| 62 | 0.66 | .421 | < .01 | .01 |
| Response Relation × Color Relation × Prime Layer × Probe Layer | 1 \| 62 | 5.60 | .021 | < .01 | .08 |

**Table 2**

*Results for the ANOVA on Error Rates, Exp. 3*

| **Effect** | **Degrees of Freedom** | ***F*-Value** | ***p*-Value** | ${\boldsymbol{\eta}_{\boldsymbol{G}}}^{\boldsymbol{2}}$ | ${\boldsymbol{\eta}_{\boldsymbol{P}}}^{\boldsymbol{2}}$ |
| --- | --- | --- | --- | --- | --- |
| Response Relation | 1 \| 62 | 1.58 | .214 | < .01 | 0.02 |
| Color Relation | 1 \| 62 | 7.32 | .009 | < .01 | 0.11 |
| Prime Layer | 1 \| 62 | 3.45 | .068 | < .01 | 0.05 |
| Probe Layer | 1 \| 62 | 7.14 | .010 | < .01 | 0.10 |
| Response Relation × Color Relation | 1 \| 62 | 17.83 | < .001 | 0.01 | 0.22 |
| Response Relation × Prime Layer | 1 \| 62 | 1.65 | .204 | < .01 | 0.03 |
| Color Relation × Probe Layer | 1 \| 62 | 0.58 | .448 | < .01 | 0.01 |
| Response Relation × Probe Layer | 1 \| 62 | 0.34 | .564 | < .01 | 0.01 |
| Color Relation × Probe Layer | 1 \| 62 | 1.58 | .213 | < .01 | 0.02 |
| Prime Layer × Probe Layer | 1 \| 62 | 5.58 | .021 | < .01 | 0.08 |
| Response Relation × Color Relation × Prime Layer | 1 \| 62 | 0.27 | .602 | < .01 | 0.00 |
| Response Relation × Color Relation × Probe Layer | 1 \| 62 | 0.20 | .659 | < .01 | 0.00 |
| Response Relation × Prime Layer × Probe Layer | 1 \| 62 | 14.45 | < .001 | 0.01 | 0.19 |
| Color Relation × Prime Layer × Probe Layer | 1 \| 62 | 0.13 | .722 | < .01 | 0.00 |
| Response Relation × Color Relation × Prime Layer × Probe Layer | 1 \| 62 | 6.28 | .015 | < .01 | 0.09 |

**Table 3**

*T-tests comparing the interaction between prime and probe layer for RT DRB effects in Exp. 3.*

| **Comparison** | **Degrees of Freedom** | ***t*-Value** | ***p*-Value** | $\boldsymbol{d}_{\boldsymbol{z}}$ | $\boldsymbol{BF}_{\boldsymbol{01}}$ |
| --- | --- | --- | --- | --- | --- |
| (Prime:) Background + (Probe:) Background vs.  Figure + Figure | 62 | -2.07 | .043 | 0.13 | 0.99 |
| Background + Background vs.  Figure + Background | 62 | 1.04 | .302 | 0.07 | 4.33 |
| Background + Background vs.  Background + Figure | 62 | 0.11 | .913 | 0.01 | 7.20 |
| Background + Figure vs.  Figure + Figure | 62 | -2.19 | .032 | 0.14 | 0.78 |
| Background + Figure vs.  Figure + Background | 62 | 0.91 | .367 | 0.06 | 4.89 |
| Figure + Background vs.  Figure + Figure | 62 | -3.05 | .003 | 0.19 | 0.11 |

**Table 4**

*T-tests against 0 for RT DRB effects as a function of prime layer and probe layer, Exp. 3.*

| **Comparison** | **Mean in ms (SD)** | **Degrees of Freedom** | ***t*-Value** | ***p*-Value** | $\boldsymbol{d}_{\boldsymbol{z}}$ | $\boldsymbol{BF}_{\boldsymbol{01}}$ |
| --- | --- | --- | --- | --- | --- | --- |
| (Prime:) Background + (Probe:) Background | 14 (39) | 62 | 2.86 | .006 | 0.36 | 0.18 |
| Background + Figure | 13 (30) | 62 | 3.49 | .001 | 0.44 | 0.03 |
| Figure + Background | 8 (32) | 62 | 2.06 | .043 | 0.26 | 1.00 |
| Figure + Figure | 27 (38) | 62 | 5.66 | < .001 | 0.71 | < 0.01 |

**Table 5**

*T-tests comparing the interaction between prime and probe layer for Error Rate DRB effects in Exp. 3*

| **Comparison** | **Degrees of Freedom** | ***t*-Value** | ***p*-Value** | $\boldsymbol{d}_{\boldsymbol{z}}$ | $\boldsymbol{BF}_{\boldsymbol{01}}$ |
| --- | --- | --- | --- | --- | --- |
| (Prime:) Background + (Probe:) Background vs.  Figure + Figure | 62 | -0.12 | .908 | 0.01 | 7.20 |
| Background + Background vs.  Figure + Background | 62 | 1.67 | .099 | 0.11 | 1.94 |
| Background + Background vs.  Background + Figure | 62 | 2.05 | .045 | 0.13 | 1.03 |
| Background + Figure vs.  Figure + Figure | 62 | -2.09 | .041 | 0.13 | 0.95 |
| Background + Figure vs.  Figure + Background | 62 | -0.75 | .458 | 0.05 | 5.55 |
| Figure + Background vs.  Figure + Figure | 62 | -2.08 | .042 | 0.13 | 0.98 |

**Table 6**

*T-tests against 0 for Error Rate DRB effects as a function of prime layer and probe layer, Exp. 3.*

| **Comparison** | **Mean in % (SD)** | **Degrees of Freedom** | ***t*-Value** | ***p*-Value** | $\boldsymbol{d}_{\boldsymbol{z}}$ | $\boldsymbol{BF}_{\boldsymbol{01}}$ |
| --- | --- | --- | --- | --- | --- | --- |
| (Prime:) Background + (Probe:) Background | 4 (9) | 62 | 3.70 | < .001 | 0.47 | 0.02 |
| Background + Figure | 1 (11) | 62 | 0.47 | .640 | 0.06 | 6.52 |
| Figure + Background | 2 (8) | 62 | 1.55 | .127 | 0.20 | 2.34 |
| Figure + Figure | 4 (8) | 62 | 4.28 | < .001 | 0.54 | < 0.01 |
